# Exercise-based rehabilitation with or without blood flow restriction in individuals with heart failure: Protocol for the rehab-washout HF trial

**DOI:** 10.1016/j.jsampl.2026.100153

**Published:** 2026-07-30

**Authors:** Robson Conceição Silva, Thaiene Martins Miranda, Lucila de Jesus Almeida, Vinícius Carvalho Fiusa, Monique Almeida Vaz, Leandro Lima de Sousa, Cristiane Koeche, Bruno Gedeon de Araújo, Viviane da Silva Machado, Andrea Stephanus, Giselle Pinto, Sergio Henrique Rodolpho Ramalho, Ana Claudia Cavalcante Nogueira, Adriana de Jesus Benevides de Almeida Guimarães, Alessandra Menezes Campos-Staffico, Hugo de Luca Corrêa, Alexandre Anderson Sousa Munhoz Soares, Carlos Ernesto Santos Ferreira, Luiz Sérgio Fernandes de Carvalho

**Affiliations:** a Postgraduate Program in Physical Education (PPGEF), Catholic University of Brasília (UCB), Brasília, Brazil; b Data Laboratory for Research on Quality of Care and Outcomes (LaDaQCOR), Catholic University of Brasília (UCB), Brasília, Brazil; c Medical Sciences Postgraduate Program (PPGCM), Universidade de Brasilia (UnB), Brasília, Brazil; d Postgraduate Program in Gerontology (PPG), Catholic University of Brasília (UCB), Brasília, Brazil; e Research Group on Cardiovascular Diseases of Brasília, Higher School of Health Sciences (ESCS), University of the Federal District (UnDF), Brasília, Brazil; f Aramari Apo Institute, Brasília, Brazil; g Department of Pharmaceutical Sciences, School of Pharmacy and Health Professions, Creighton University, Omaha, USA; h Department of Cardiology, State University of Campinas (Unicamp), Campinas, Brazil; i Clarity – Health Intelligence, Campinas, Brazil; j Marinha o Brasil, Brazil; k Internal Medicine and Cardiology Services, Hospital Sírio-Libanês, Brasília, Brazil

**Keywords:** Heart failure, Resistance training, Blood flow restriction, Cardiorespiratory fitness, Biomarkers

## Abstract

**Background:**

Heart failure (HF) significantly reduces functional capacity and quality of life in patients classified as New York Heart Association (NYHA) functional class II–III. While cardiopulmonary rehabilitation (CR) is beneficial, innovative strategies are required to improve accessibility and effectiveness for individuals unable to tolerate high hemodynamic loads.

**Aims:**

To compare the effects of conventional moderate-to-high intensity resistance training (CRT) versus low-load resistance training combined with blood flow restriction (BFRRT) on clinical, functional, and biochemical outcomes in HF patients.

**Methods and analysis:**

This randomized, prospective, parallel-group clinical trial will include 52 patients (NYHA II–III) allocated into CRT (60–80% 1RM) or BFRRT (20–30% 1RM, calibrated at 50% arterial occlusion pressure) groups. The supervised intervention lasts 4 months, with an 8-month post-intervention follow-up. The primary outcome is the change in serum NT-proBNP levels. Secondary outcomes include quality of life, echocardiographic ventricular function, functional capacity, and clinical events. Advanced statistical models and exploratory machine learning analyses with k-fold cross-validation will be utilized for predictive modeling and exploring personalized medicine strategies.

**Implications:**

The REHAB-WASHOUT HF trial aims to establish an effective, safe, and accessible therapeutic approach for HF rehabilitation, potentially enhancing clinical outcomes and guiding personalized prescriptive strategies in cardiovascular rehabilitation.

**Ethics and registration:**

Approved by the local institutional review board (No. 5.848.965; CAAE 64955022.9.1001.0029). Trial registration: RBR-5y8k7d7.

## Highlights

• REHAB-WASHOUT HF trial evaluates blood flow restriction in heart failure patients.
• It compares moderate-to-high load training against low-load BFR training.
• The primary outcome is the change in serum NT-proBNP levels at 4 months.
• A strict safety monitoring and session stopping criteria framework is detailed.
• Machine learning with cross-validation will identify response predictors.

## 1 Introduction

Heart Failure (HF) imposes a substantial burden on healthcare systems, generating costs exceeding US$100 billion annually and affecting more than 64 million people worldwide [1,2]. Although advanced pharmacological strategies—such as Angiotensin Receptor-Neprilysin Inhibitors and Sodium-Glucose Cotransporter 2 Inhibitors—have recently entered the market, the quality of life of patients with HF remains severely impaired, particularly in low-access and low-socioeconomic status contexts [3].

**Table 1:** Schedule of enrollment, interventions, and assessments (REHAB-WASHOUT HF).

Phase	Enrollment	Baseline (T0)	Adaptation & 1RM	Intervention (16 weeks)	Post-intervention (T1)	Follow-up (T2)
**Timepoint**	Weeks −3 to −2	Weeks −2 to −1	Weeks 1–3	Weeks 4–15	Week 16 (Month 4)	Month 12
**ENROLLMENT**						
Eligibility screening	X					
Informed consent	X					
Randomization		X				
**INTERVENTIONS**						
Adaptation (CRT only)			X			
Supervised exercise (2×/wk)				X		
Unsupervised activity counseling					X	X
**ASSESSMENTS**						
Clinical (DXA, skinfolds, ABI)		X			X	X
Biochemical (BNP/NT-proBNP, microRNA)		X			X	X
Functional (CPET, 6MWT, sit-to-stand)		X			X	X
Imaging (transthoracic echocardiogram)		X			X	X
Strength (1RM test and retest)		X	X	X (adjustment)	X	X
Strength (isokinetic & handgrip)		X			X	X
Quality of life (Minnesota MLHFQ)		X			X	X
Clinical events & hospitalizations				X	X	X

**Fig. 1:**
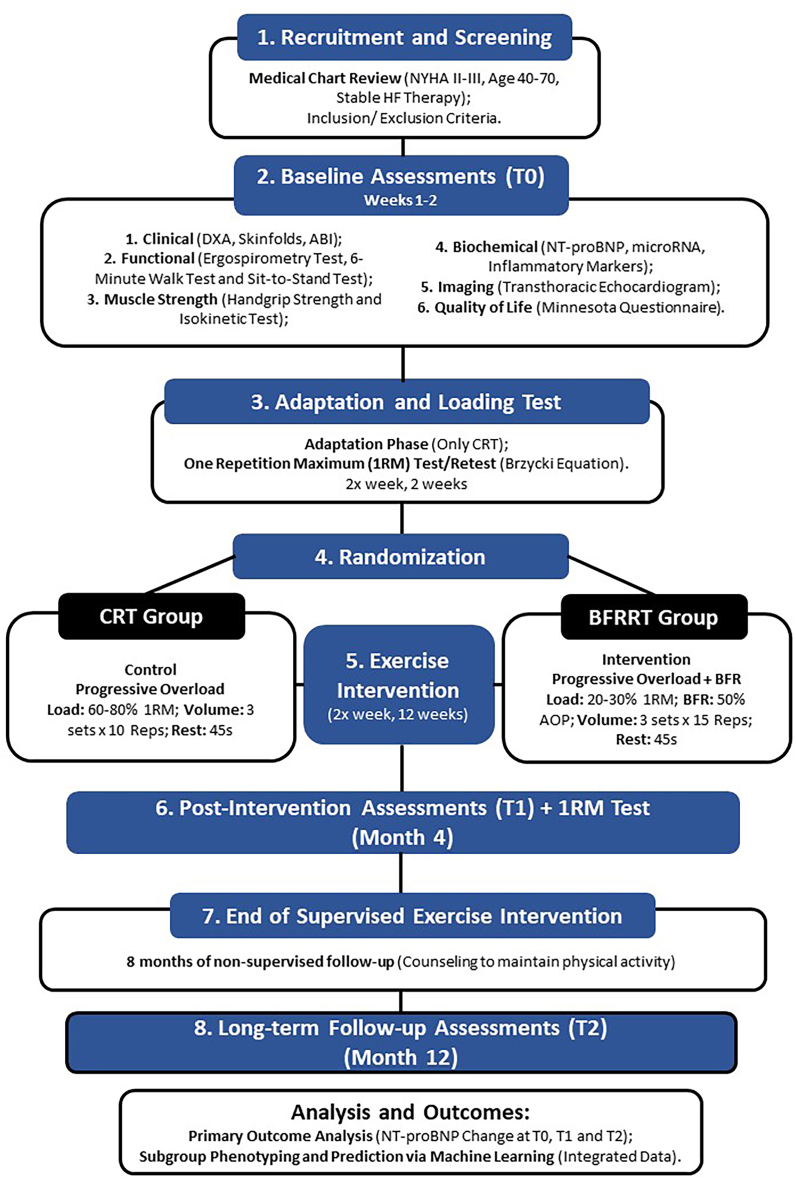
Flow chart of study procedures.

Currently, cardiopulmonary rehabilitation (CR) holds a class I recommendation in guidelines from both the European Society of Cardiology and the American College of Cardiology [4,5]. Despite the established therapeutic role of physical training in HF populations, the heterogeneity of exercise interventions limits the consensus on the most effective prescription [6]. Furthermore, the limited global availability of structured rehabilitation programs negatively impacts patient outcomes and quality of life [7,8].

In part, the severe exercise intolerance observed in these patients reduces adherence and compromises the expected prognosis in conventional programs [9]. Traditional resistance training (CRT) typically requires moderate-to-high external loads (60–80% of 1-repetition maximum [1RM]) to induce muscular hypertrophy and strength gains. However, such intensities induce elevated central hemodynamic stress, which is often intolerable or contraindicated for individuals with advanced HF [10].

To overcome these limitations, resistance training combined with blood flow restriction (BFRRT) has emerged as a promising clinical strategy, having demonstrated safety and efficacy in other cardiovascular populations such as coronary artery disease and hypertension. By applying partial vascular occlusion to the working limbs, BFRRT allows the use of low external loads (20–30% 1RM) while mimicking the metabolic stress and hypertrophic stimuli of high-load training, thereby minimizing central cardiac overload [11,12]. However, evidence regarding BFRRT specifically in heart failure remains limited.

Therefore, this study aims to compare the effects of low-intensity resistance training versus resistance training with blood flow restriction (BFRRT) on clinical and functional outcomes in patients with HF. The primary objective is to evaluate the progression of serum N-terminal pro-brain natriuretic peptide (NT-proBNP) levels before and after a 4-month intervention. As secondary objectives, this trial will assess the impact of these protocols on health-related quality of life, functional capacity, muscular strength, left and right ventricular function, and clinical adverse events. The clinical impact will be monitored over 12 months, comprising a 4-month supervised exercise intervention with differing loading intensities followed by an 8-month period of physical activity maintenance at home with subsequent reassessment. Overall, this trial will provide novel insights into the feasibility, safety, and physiological adaptations of BFR in heart failure, establishing an accessible therapeutic paradigm for cardiovascular rehabilitation.

## 2 Methods

### 2.1 Study design and ethical approvals

This is a single-center, prospective, randomized, parallel-group, open-label clinical trial protocol designed to compare the effects of two cardiopulmonary rehabilitation (CR) protocols—resistance training with versus without blood flow restriction (BFRRT)—in patients with heart failure (HF).

This study protocol is structured and will be reported in accordance with the Standard Protocol Items: Recommendations for Interventional Trials (SPIRIT) guidelines. The study protocol received ethical approval from the Institutional Review Board of the Catholic University of Brasília (UCB) (Brasília, Brazil) under protocol number CAAE 64955022.9.1001.0029.

Written informed consent will be obtained from all participants prior to their formal inclusion in the trial. The investigation will be conducted in strict accordance with the ethical principles outlined in the Declaration of Helsinki (Table 1).

### 2.2 Sample size calculation

The sample size calculation was informed by the data reported by Tanaka et al. [13], who evaluated a BFR-based exercise protocol in patients with HF (New York Heart Association [NYHA] functional class I or II). In their trial, a post-training reduction in brain natriuretic peptide (BNP) of 73 ± 68 pg/mL was observed in the BFR group, compared to a reduction of 15 ± 70 pg/mL in the conventional CR control group [13].

Based on these values, a Cohen's *d* effect size of 0.83 was calculated. Assuming a 1:1 allocation ratio between the experimental arms, a minimum of 24 patients per group is required to achieve a statistical power of 80% with a two-tailed alpha level of 0.05. To account for an anticipated attrition rate of approximately 10%, the final sample size has been established at 26 patients per treatment arm, resulting in a total target recruitment of 52 participants. BNP data from Tanaka et al. were used as the closest available proxy because both biomarkers reflect cardiac wall stress and no directly comparable BFR exercise trial reporting NT-proBNP was available, although this trial will evaluate N-terminal pro-brain natriuretic peptide (NT-proBNP) as its primary biochemical outcome.

### 2.3 Participants and recruitment

Participants will be recruited from the outpatient cardiology clinics of the Catholic University of Brasília (UCB) and the State Department of Health of the Federal District (SES−DF), including primary healthcare units, the Gama Regional Hospital (HRG), and the Guará Regional Hospital (HRGu). Medical records will be screened prospectively to identify potentially eligible candidates (Figure 1).

### 2.4 Eligibility criteria

Eligibility criteria will be applied through a rigorous multi-stage screening process based on the following inclusion and exclusion parameters.

#### 2.4.1 Inclusion criteria

Patients aged between 40 and 70 years; Documented diagnosis of heart failure with reduced ejection fraction (HFrEF) or heart failure with preserved ejection fraction (HFpEF); Classification within the New York Heart Association (NYHA) functional class II or III; Optimization of stable medical therapy for at least 6 weeks prior to enrollment, which must include a beta-blocker combined with either an angiotensin-converting enzyme inhibitor, an angiotensin receptor blocker, or an angiotensin receptor-neprilysin inhibitor.

#### 2.4.2 Exclusion criteria

Any absolute or relative contraindication to exercise testing or training according to international guidelines; History of a major cardiovascular event (e.g., acute myocardial infarction, unstable angina) or cardiovascular procedures (e.g., percutaneous coronary intervention, coronary artery bypass grafting) within the 2 months preceding enrollment; Implantation of a fixed-rate pacemaker where the upper heart rate limit is programmed below the target heart rate calculated for physical training; Specific contraindications to blood flow restriction therapy, including a documented history of deep vein thrombosis (DVT), peripheral vascular disease, active thrombophlebitis, or severe peripheral neuropathy; Inability to perform resistance training due to severe orthopedic or cognitive limitations.

### 2.5 Primary and secondary outcomes

#### 2.5.1 Primary outcome

The primary outcome of this trial will be the longitudinal change in serum N-terminal pro-brain natriuretic peptide (NT-proBNP) levels from baseline to the completion of the 16-week intervention. Serum NT-proBNP is a gold-standard, objectively measured biomarker reflecting myocardial wall stress and hemodynamic overload. Reductions in its concentration serve as a surrogate marker for positive cardiac reverse remodeling and are strongly correlated with reduced long-term cardiovascular mortality and hospitalization rates in individuals with heart failure.

#### 2.5.2 Secondary outcomes

To capture a comprehensive clinical profile of the chronic adaptations induced by the training protocols, the following secondary outcomes will be evaluated at baseline and after 16 weeks:

Health-Related Quality of life: Assessed using the Minnesota Living with Heart Failure Questionnaire. This specific instrument evaluates the patients' perceptions of how the disease affects their physical, socioeconomic, and emotional dimensions, acting as a powerful clinical predictor of disease stability and patient independence.

Functional Capacity and Exercise Tolerance: Measured via the distance covered in the 6-Minute Walk Test. Functional capacity is an independent predictor of all-cause mortality in heart failure, and its improvement directly relates to real-world functional independence.

Peripheral Muscular Strength: Evaluated through the submaximal one-repetition maximum 1RM prediction protocol. Skeletal muscle wasting and weakness are core features of the pathophysiological progression of heart failure; enhancements in peripheral muscular strength are strongly linked to improved mechanical efficiency and reduced clinical frailty.

Echocardiographic Ventricular Function: Left and right ventricular structural and functional remodeling will be assessed via resting transthoracic echocardiography. Key parameters include left ventricular ejection fraction (LVEF), tricuspid annular plane systolic excursion (TAPSE), and diastolic filling pressures (*E/e'* ratio) to document central cardiac adaptations.

Clinical Safety and Adverse Events: The occurrence of any relative or absolute adverse event (e.g., symptomatic hypotension, persistent muscular pain, dizziness, or pre-syncope), all-cause hospitalizations, or clinical worsening of the heart failure condition will be systematically recorded throughout the 16-week cycle to establish safety parameters.

#### 2.5.3 Randomization and allocation concealment

Following the completion of baseline assessments, participants will be randomly assigned (1:1) to either the Conventional Resistance Training (CRT) group or the Blood Flow Restriction Resistance Training (BFRRT) group. Randomization will be executed using an automated, computer-generated sequence with a permuted block design (block size of 2) to ensure a strictly balanced allocation between the treatment arms.

To maintain strict allocation concealment, the randomization sequence will be generated and managed by an independent research coordinator who is not involved in participant recruitment, daily interventions, or outcome evaluations. Individual group assignments will be enclosed in sequentially numbered, opaque, sealed envelopes, which will be opened only after the participant completes all baseline evaluations.

#### 2.5.4 Exercise intervention protocol

The structured cardiopulmonary rehabilitation (CR) program will span a 16-week period, with sessions conducted twice weekly, each lasting approximately 30 min. The initial 2 weeks of the program will serve as a standardized adaptation phase, during which all participants across both experimental arms will perform low-load conventional resistance training.

In the third week, a one-repetition maximum (1RM) test and retest procedure will be executed to establish baseline dynamic muscular strength for training load prescription. To minimize the risk of cardiovascular adverse events and avoid maximal intra-thoracic pressure or prolonged Valsalva maneuvers in this clinical population, maximum muscular strength will be indirectly estimated via submaximal testing using the Brzycki prediction equation [14]:

$$1\text{RM}=\frac{\text{Load}}{\left(1.0278\;–\;\left(0.0278\;\times \;\text{Reps}\right)\right)}$$

Where Load represents the submaximal weight lifted (expressed in kilograms) and Reps denotes the number of repetitions completed until volitional fatigue, not exceeding 10 repetitions. This submaximal 1RM estimation protocol will be performed for all selected exercises at baseline to calculate the exact relative percentages for training prescription, and it will be repeated during the final week of the 16-week intervention to evaluate muscular strength progression as a secondary outcome.

#### 2.5.5 Training cycles and periodization progression

The structured intervention within the allocated groups (CRT or BFRRT) will commence in the fourth week of the program. The macrocycle will be partitioned into two distinct 6-week training blocks. To ensure appropriate progressive overload, a submaximal 1RM reassessment will be conducted at the end of the first 6-week block to recalibrate relative training loads. A final submaximal 1RM evaluation will be executed during the sixteenth week to analyze long-term muscular strength progression.

#### 2.5.6 Session structure and exercise selection

Each training session will initiate with a 5-min quiet rest period in the seated position for baseline hemodynamics and blood pressure monitoring. Participants will then perform a 5-min warm-up on a motorized treadmill at a self-selected comfortable pace, followed immediately by the resistance training circuit.

The circuit will comprise the following five exercises targeted at major muscle groups:

Horizontal leg press; Seated chest press (bench press machine); Seated lat pulldown; Leg curl; Standing calf raises (plantar flexion). Following session completion, a 5-min passive recovery period will be observed, followed by final blood pressure and heart rate measurements.

#### 2.5.7 Group-specific interventions and loading conditions

To isolate the therapeutic effect of vascular occlusion, the structural variables—including the number of sets, repetitions, and rest intervals—will be identical between groups, whereas the external training intensities differ by design: Conventional Resistance Training (CRT) Group: Participants will perform 3 sets of 15 repetitions at a moderate-to-high intensity of 60–80% of 1RM, maintaining a 45-s passive rest interval between sets. Blood Flow Restriction Resistance Training (BFRRT) Group: Participants will execute 3 sets of 15 repetitions at a low external intensity of 20–30% of 1RM, maintaining a 45-s passive rest interval. For this group, blood flow restriction will be applied continuously throughout both the active exercise bouts and the brief inter-set rest periods, and it will be deflated immediately upon completion of the third set of each exercise.

#### 2.5.8 Vascular occlusion pressure calibration

For the BFRRT group, the total arterial occlusion pressure (AOP) will be determined in the fourth week and reassessed alongside the submaximal 1RM tests to account for anatomical and localized adaptations. Measurements will be performed by a trained investigator using a portable vascular Doppler probe (DV 610 B, Medmega, Brazil) to detect the cessation of the auscultatory pulse.

Vascular occlusion will be applied using a clinical blood flow restriction system equipped with a removable aneroid manometer and high-resistance velcro cuffs (Avanutri, Rio de Janeiro, Brazil). To standardize application, the baseline AOP measurement will be performed while the patient is positioned in a relaxed, standing orthopedic posture (anatomical position). The restriction pressure during the sessions will be dynamically calibrated and monitored in real time via the integrated aneroid gauge, set at a standardized, individualized target of 50% of the total AOP for both the upper and lower limbs. The cuffs will remain continuously inflated throughout the specific exercise execution and its corresponding 45-s inter-set rest intervals; however, the cuffs will be completely deflated immediately upon completion of the final set of each exercise and will remain deflated during transitions between different exercise stations to ensure patient safety and comfort.

#### 2.5.9 Procedures

During the first two weeks, participants will undergo comprehensive baseline clinical evaluations. Body composition will be assessed via dual-energy X-ray absorptiometry (DXA) as the primary laboratory standard; additionally, skinfold thickness measurements will be collected as a methodological safeguard and field-based backup to ensure data continuity throughout the long-term trial. Vascular health, cardiorespiratory fitness, and functional performance will be evaluated using the ankle-brachial index, cardiopulmonary exercise testing (ergospirometry), the 6-min walk test, and the 30-s sit-to-stand test.

Muscle strength will be assessed through knee-joint isokinetic dynamometry and handgrip strength. Furthermore, biochemical analyses will be performed to measure blood-borne biomarkers: hemostasis markers (D-dimer, fibrinogen, and activated partial thromboplastin time—aPTT) will be monitored as a clinical safety protocol, alongside systemic inflammatory markers (C-reactive protein) and tissue-specific microRNAs (vascular, skeletal muscle, and cardiac-specific).

Cardiac structure and function will be analyzed via resting transthoracic echocardiography, while health-related quality of life will be measured using the Minnesota Living with Heart Failure Questionnaire.

#### 2.5.10 Dual-energy X-ray absorptiometry

Body composition will be assessed via whole-body Dual-Energy X-ray Absorptiometry (DXA), using the DPX-IQ model (Lunar, Madison, USA), which will be properly calibrated daily according to the manufacturer's operational recommendations. Prior to the scanning procedure, participants will be instructed to remove all metallic accessories, jewelry, and clothing components containing metal to prevent X-ray attenuation artifacts.

During the diagnostic scan, participants will remain completely immobile in the supine position on the central axis of the equipment table. Their arms will be positioned minimally away from the trunk with palms facing downward, and their feet will be secured apart to standardize tracking boundaries. For safety compliance, individuals fitted with permanent cardiac pacemakers or Implantable Cardioverter Defibrillators (ICDs) will be strictly excluded from performing this specific imaging test.

#### 2.5.11 Skinfold thicknesses

To evaluate subcutaneous body fat distribution, skinfold thicknesses will be collected across the following nine standardized anatomical sites: biceps, triceps, subscapular, pectoral, midaxillary, suprailiac, abdominal, medial thigh, and calf. All anthropometric markings and localized tissue caliper placements will be executed in strict accordance with the morphological procedures outlined by the American College of Sports Medicine (ACSM) Guidelines for Exercise Testing and Prescription, 11th Edition [15].

These manual measurements will be performed unilaterally on the right side of the body by a single, experienced investigator to eliminate inter-rater variability. The evaluator will maintain a verified test-retest reliability coefficient greater than 0.95 for each separate anatomical site and a maximum technical error of measurement (TEM) restricted to ±1.0 mm, utilizing a calibrated Lange skinfold caliper. All physical measurements will be captured using a rotational tracking order, repeated three separate times per site, and logged by an independent data recorder. The median value of the three consecutive trials for each anatomical landmark will be adopted as the formal baseline reference.

Total body density will be calculated from the cumulative skinfold thickness arrays utilizing generalized predictive equations validated for adult and older clinical cohorts. The Jackson and Pollock 7-skinfold mathematical protocol will be deployed for both male and female configurations; this specific model is selected because it actively incorporates chronological age as a corrective biological variable, which is critical for accurate body composition tracking within the targeted 40–70 age spectrum [15]. Subsequently, the estimated body density metrics will be converted to isolate the final relative body fat percentage using the standard Siri formulation [16].

#### 2.5.12 Ankle-brachial index

The volunteer will remain at rest, lying supine on a stretcher for 5 min in a quiet environment with a temperature around 25 °C. After that, blood pressure will be measured in the arms and legs, positioning the cuffs of automatic blood pressure devices (BP3AC1, Microlife, Shenzhen, China) approximately 2 cm above the cubital fossa and 2 cm above the malleoli, respectively. After placing the cuffs, blood pressure will be measured simultaneously, first on the right side and then on the left side [17].

The Ankle-Brachial Index (ABI) calculation will be performed using the following formula: ABI = Highest Ankle Systolic Blood Pressure/Highest Arm Systolic Blood Pressure. After determining the ABI, patients will be classified as normal ABI: 0.91 to 1.30) and abnormal ABI: ≤0.90 or >1.30 [17].

#### 2.5.13 Ergospirometry test

The maximal cardiopulmonary exercise test (CPET) will be performed on a motorized treadmill (Millennium ATL, Imbramed, Porto Alegre, Brazil) utilizing an individualized ramp protocol [18], designed to elicit volitional exhaustion within 8–12 min. The primary objective is to evaluate electrocardiographic and hemodynamic responses, alongside peak oxygen consumption (VO2 peak), during progressive physical exertion. Continuously recorded 3-lead electrocardiographic signals (CM5, D2M, and V2M) will be captured via a portable digital electrocardiograph (MEBT-100, Micromed, Brazil) using solid-gel silver/silver-chloride adhesive electrodes (Kendall, Cardinal Health 200, USA) affixed to the patient's chest following thorough skin preparation with gauze and 70% isopropyl alcohol.

Systolic and diastolic blood pressures will be monitored minute-by-minute using a calibrated manual aneroid sphygmomanometer (Welch Allyn, Chicago, IL, USA). To quantify metabolic gas exchange, a breath-by-breath automated respiratory gas analyzer (Cortex Metalyzer, Leipzig, Germany) will be deployed, with metabolic data continuously aggregated and recorded every 10 s.

Participants will receive strict pre-test instructions to arrive at the laboratory well-hydrated, appropriately nourished, and wearing athletic clothing. Consumption of caffeine-containing foods or beverages and tobacco smoking will be strictly prohibited for at least 2 h preceding the evaluation, and strenuous physical exertion will be restricted for 24 h prior to testing. Additionally, male participants will undergo chest hair trichotomy at the electrode placement sites to maximize signal quality.

For standardization, all evaluations will be scheduled during morning hours and will be continuously supervised by a clinical cardiologist. To ensure patient safety, the test will be immediately terminated upon volitional exhaustion, or prior if absolute clinical indications for termination manifest, including progressive drops in systolic blood pressure, severe chest pain indicative of angina, complex ventricular arrhythmias, or horizontal/downsloping ST-segment depression greater than 2.0 mm [19].

#### 2.5.14 6-Minute walk test

The test will be performed to assess submaximal aerobic endurance and functional walking capacity. The testing course will be set up in a long, flat corridor where two cones will be placed 30 m apart, with distinct visual markings positioned every 5 m between them. Participants will be instructed to walk continuously around the course for 6 min, attempting to cover the maximum possible distance without running or jogging. A trained investigator will be responsible for tracking and recording the total distance covered, providing standardized phrases of encouragement and announcing the remaining time every minute. If severe dyspnea or fatigue manifests, participants will be permitted to stop, rest on a chair, and resume walking as soon as they feel capable [20].

#### 2.5.15 Sit-to-stand test

The test will be performed to assess the functionality, power, and muscle endurance of the lower limbs, in addition to being an indicator of mobility and functional balance. At the command of “attention, go!”, volunteers will sit down and stand up from a chair (without the aid of the upper limbs) as many times as they can in 30 s [20].

The test will start with the volunteer seated, with their back supported, feet shoulder-width apart (fully supported on the floor), and upper limbs crossed. When standing, they will fully extend the trunk, and when returning to the starting position, they will fully rest their buttocks on the seat. A researcher will observe the test and count only correct executions. Verbal guidance will be provided to correct incorrect executions. A chair without arms, with a seat height of approximately 43 cm will be used, and for safety reasons, it will remain supported against a wall [20].

#### 2.5.16 Isokinetic test

To measure the isokinetic muscle strength and power of the quadriceps and hamstrings, the Biodex System 3 isokinetic dynamometer (Biodex Medical Systems, New York, USA) will be used. Before each test, participants will warm up on a cycle ergometer (Ergomedic 828 E, Monark, Vansbro, Sweden) for 5 min with a workload of 50 W [21]. The dynamometer configurations and axis alignment will be adjusted as suggested by Stumbo et al. (2001) [22].

During the test, participants will be secured with the equipment stabilization belts to minimize cooperation from other non-specific muscle groups [22]. At the command of “attention, go!”, participants will perform five maximal repetitions of knee extension and flexion using the maximum possible force, maintaining a standardized range of motion of 70°. The test will be performed on both legs in a concentric/concentric mode, utilizing two angular velocities (60° per second and 180° per second) with a 1-min recovery interval between them.

All participants will be vigorously encouraged verbally during the execution. The primary outcome variables extracted from the software will comprise peak torque, total work, and power.

#### 2.5.17 Handgrip strength

The test will be measured with a JAMAR hydraulic handgrip dynamometer (Sammons Preston Inc., Illinois, USA). During the test, participants will be seated in an armless chair with an erect trunk posture, the elbow flexed at 90°, and the forearm maintained in a neutral semi-pronation position. The grip on the dynamometer will be individually adjusted according to the size of the hands, ensuring the inner bar closest to the body of the dynamometer remains positioned precisely on the second phalanges of the index, middle, and ring fingers [23].

Two measurements will be taken on each hand alternately, respecting a 1-min resting interval between attempts. The test will start with the command “attention, go!”, and participants will squeeze the dynamometer gradually until reaching absolute maximum force, avoiding any compensatory radial or ulnar wrist deviation. The highest peak force value reached in each hand will be considered as the definitive reference metric.

#### 2.5.18 Biochemical tests blood

Blood samples will be collected via venipuncture after a strict 12-h overnight fast. All laboratory analyses will be executed at the central facility of the Catholic University of Brasília. Approximately 28 aliquots per participant will be prepared for immediate storage in liquid nitrogen at the facility, and 14 aliquots will be stored in ultra-low temperature freezers at minus 80 °C at the research center for future exploratory biomarker analyses.

The comprehensive laboratory panel will include whole blood cell count determined via an automated counter method. Metabolic and lipid profiles will comprise fasting plasma glucose, total cholesterol, high-density lipoprotein cholesterol, and triglycerides, all analyzed using the ADVIA Chemistry system (Siemens, Deerfield, Illinois). Low-density lipoprotein cholesterol will be calculated utilizing the Friedewald equation, or measured directly if triglyceride levels exceed 400 mg per deciliter.

To evaluate systemic inflammatory responses, organ function, and clinical safety markers, the panel will analyze high-sensitivity C-reactive protein and fibrinogen (Dade Behring; Siemens); creatinine, uric acid, calcium, gamma-glutamyl transferase, aspartate aminotransferase, and alanine aminotransferase (ADVIA Chemistry); glycated hemoglobin (Bio-Rad Laboratories, Hercules, California); and creatine phosphokinase to monitor skeletal muscle integrity.

Endocrine regulation and cardiac strain will be determined via thyroid-stimulating hormone, thyroxin, insulin, and C-peptide (Siemens). Crucially, N-terminal pro-brain natriuretic peptide (Dade Behring; Siemens) will be quantified as a primary biomarker of myocardial wall stress, and microalbuminuria (Dade Behring) will be assessed to track baseline microvascular renal function. These biochemical markers will be utilized to ensure patient safety compliance regarding renal and hepatic thresholds prior to exercise, and to serve as clinical baseline covariates.

#### 2.5.19 Transthoracic echocardiogram

Echocardiographic images will be acquired according to the clinical recommendations of the American Society of Echocardiography using a commercially available Philips Affiniti 70 ultrasound system (Philips Ultrasound, Andover, Massachusetts, USA) equipped with a X5-1 transducer operating between 1 and 5 MHz.

The assessment of diastolic dysfunction and reduced left ventricular ejection fraction (defined as less than or equal to 54 percent for women and less than or equal to 52 percent for men) will be characterized according to current consensus guidelines [24,25]. Left ventricular end-diastolic diameter, interventricular septum thickness, and left ventricular posterior wall thickness will be measured at end-diastole. Left ventricular mass will be calculated according to the Devereux formula [24].

The left ventricular mass index will be calculated by dividing the total left ventricular mass by the body surface area, with the latter determined using the Mosteller formula [26]. Ventricular hypertrophy will be defined as a left ventricular mass index greater than or equal to 102 g per square meter for men and greater than or equal to 88 g per square meter for women [24]. Three consecutive cardiac cycles of all images obtained in the apical axes (four-chamber, two-chamber, and three-chamber views) will be acquired, alongside targeted recordings from the apical four-chamber windows focused specifically on the right ventricle [27]. These recordings will serve to perform advanced deformation analysis of the left ventricular global longitudinal strain, right ventricular free wall strain, and left atrial strain.

To optimize the echocardiographic assessment via speckle tracking, images will be captured at a high temporal resolution of 40–70 frames per second [28]. Subsequently, these digital cardiac cycles will be transferred to a dedicated QLAB workstation (version 10.2, Philips, the Netherlands) for multi-chamber strain analysis. Peak longitudinal regional strain will be measured across the 17 myocardial segments of the left ventricle, and a weighted average will be computed to determine the final left ventricular global longitudinal strain value [25]. Left ventricular global longitudinal strain values higher than minus 16 percent (meaning lower than 16 percent in absolute value) will be classified as abnormal [28]. Right ventricular longitudinal strain will be analyzed utilizing the right ventricle-focused apical four-chamber view, and the average strain value will be obtained from the three anatomical segments of the right ventricular free wall [29]. A free wall right ventricular longitudinal strain value higher than minus 20 percent (lower than 20 percent in absolute value) will be considered abnormal, complying with the 2015 European Association of Cardiovascular Imaging and American Society of Echocardiography chamber quantification guidelines and the recent multinational World Advisory Strain Evaluation study [25,26,30].

Left atrial strain analysis will be executed using the dedicated left atrial automated function imaging software package and will be based on both the left atrium-focused four-chamber and two-chamber views [31]. The total left atrial reservoir strain, which possesses the largest body of clinical evidence supporting its prognostic utility in heart failure, will be reported [32]. The lower normal limit for the left atrial reservoir strain will be set at 16.7 percent [31]. Importantly, these multi-chamber echocardiographic metrics will be utilized to robustly characterize the phenotypic heart failure profiles of the sample and to strictly exclude patients presenting with severe uncorrected valvular diseases or obstructive hypertrophic cardiomyopathy, ensuring absolute hemodynamic safety prior to the exercise protocols.

#### 2.5.20 Quality of life questionnaire

The Minnesota Living with Heart Failure Questionnaire will be administered by the same trained investigator before and immediately after the 16-week cardiovascular rehabilitation protocol, utilizing the instrument validated for the Brazilian population [33]. This disease-specific questionnaire consists of 21 individual items designed to measure the patients' perceptions of the effects of heart failure on their physical, socioeconomic, and psychological lives.

Each question is scored on a 6-point Likert scale ranging from 0 (indicating no limitation) to 5 (indicating maximum limitation). The items are structurally divided into three distinct dimensions: a physical dimension, an emotional dimension, and a generalized dimension incorporating financial considerations and lifestyle constraints. The total cumulative score will range from 0 to 105 points, with a lower overall score reflecting a superior disease-specific quality of life.

The cutoff points used to categorize the participants' quality of life status will be defined as follows: scores up to 26 points indicate a good quality of life; scores from 26 to 45 points indicate a moderate quality of life; and scores higher than 45 points signify a poor quality of life [33,34].

#### 2.5.21 Statistical analysis and machine learning plan

All primary and secondary clinical, functional, and biochemical outcome analyses will be conducted strictly based on the intention-to-treat principle. Accordingly, all randomized participants will be evaluated within their originally allocated treatment groups, regardless of compliance, session attendance, missing data, or subsequent protocol deviations. Continuous variables will be presented as mean plus or minus standard deviation for data with a normal distribution, or as median and interquartile range for non-normally distributed data.

Categorical variables will be expressed as absolute frequencies and percentages and will be compared utilizing the chi-square test. Continuous variables demonstrating normal distribution will be analyzed using analysis of covariance models, adjusted for age and sex as clinical baseline covariates. The essential prerequisites for the analysis of covariance models—including linearity, normality of distribution, and homogeneity of variance—will be strictly verified through visual inspection of histograms, normal probability plots, and residual scatter plots.

Continuous variables with non-normal distribution will undergo logarithmic transformation to correct distribution asymmetry. If normal distribution is not achieved following transformation, generalized linear models or robust statistical methods will be applied to guarantee appropriate adjustments for age and sex. All classical statistical analyses will be conducted utilizing the R Studio environment (version 1.12, R language version 4.2).

In addition to traditional frequentist statistical analyses, advanced machine learning and artificial intelligence techniques will be deployed to optimize data interpretation and discover predictive biomarkers of therapeutic response to the physical exercise interventions. To predict individual clinical responses to the cardiovascular rehabilitation protocol—specifically changes in N-terminal pro-brain natriuretic peptide, peak oxygen consumption, and quality of life scores—supervised learning algorithms will be implemented, including random forest, gradient boosting machines, and support vector machines. To mitigate the risk of model overfitting and ensure generalizability, these supervised algorithms will be evaluated using a k-fold cross-validation strategy.

Unsupervised learning methods, specifically k-means and hierarchical clustering algorithms, will be utilized to perform clinical phenotyping of patient subgroups based on multidimensional profiles incorporating baseline inflammatory, functional, and echocardiographic variables. Feature selection and model interpretability will be enhanced using least absolute shrinkage and selection operator regression alongside Shapley Additive exPlanations values to identify and rank the most relevant predictive clinical variables. Longitudinal data tracking dynamic trajectories of clinical response over time will be comprehensively analyzed using mixed-effects models and, where applicable, recurrent neural networks.

## Ethical compliance

The study protocol was reviewed and approved by the Research Ethics Committee of the Catholic University of Brasília (CAAE: 64955022.9.1001.0029). All procedures were conducted in accordance with the ethical principles outlined in the Declaration of Helsinki. Written informed consent was obtained from all participants prior to their inclusion in the study.

## Declaration of generative AI and AI-assisted technologies in the manuscript preparation process

During the preparation of this work the author(s) used Gemini (Google) in order to improve the English language, technical terminology, and manuscript formatting. After using this tool/service, the author(s) reviewed and edited the content as needed and take(s) full responsibility for the content of the published article.

## Funding

This study was supported by the Fundação de Apoio à Pesquisa do Distrito Federal (FAPDF, Research Support Foundation of the Federal District) [Grant number: 00193-00001814/2023-18]. The funding source had no role in study design, in the collection, analysis and interpretation of data, or in the decision to submit the article for publication.

## Declaration of competing interest

The authors declare that they have no known competing financial interests or personal relationships that could have appeared to influence the work reported in this paper.
